# Food insecurity confrontation by pastoralist and agrarian communities in South Omo Zone, Ethiopia: a facility-based qualitative study

**DOI:** 10.1017/jns.2024.88

**Published:** 2025-01-09

**Authors:** Mintesinot Melka Gujo, Lebitsi Maud Modiba

**Affiliations:** 1 South Ethiopia Region Health Bureau Public Health Institute, Jinka, Ethiopia; 2 Department of Health Studies, College of Human Sciences, University of South Africa, Pretoria, South Africa

**Keywords:** Agrarian, Confrontation, Coping mechanisms, Food insecurity, Pastoralist, AIDS, Acquired immunodeficiency syndrome, FGD, Focus group discussions, FAO, Food and Agriculture Organization, GHI, Global Hunger Index, SDGs, Sustainable Development Goals, SSA, Sub-Saharan Africa, UNISA, University of South Africa

## Abstract

Food insecurity remains a global issue, particularly in developing countries like Ethiopia. Thus, this study focused on identifying factors contributing to food insecurity and the strategies used to cope with it among agrarian and pastoralist communities of South Ari and Benatsemay Woreda, respectively. A facility-based qualitative study was carried out in Southern Ethiopia. Participants were selected using a purposefully targeting health extension workers, health centre directors, woreda programme experts, district health managers, and pregnant women staying in maternity waiting homes. The selection process included one health facility from each district, focusing on those with the highest number of pregnant women in maternity waiting homes. A total of 17 participants were involved in in-depth interviews, and 2 focus group discussions were conducted with 27 pregnant women, continuing until data saturation was achieved. Field notes were taken, and sessions were voice recorded. Participants in both in-depth interviews and focus group discussions frequently identified several causes of food insecurity in the community, such as food shortages, climate change, rising prices of agricultural products, inadequate agricultural technology, scarcity of farmland, and income constraints. Tailored intervention is highly demanding to implement policies to stabilise food supply chains and mitigate food shortages in both agrarian and pastoralist areas, invest in modern agricultural technologies to boost productivity, encourage the adoption of climate-smart agricultural practices to help farmers adapt to changing weather patterns, optimise the productive use of available farmland, promote income-generating activities, and diversify livelihoods to alleviate income constraints and improve food security.

## Introduction

Food insecurity refers to a situation where individuals face restricted access both physically and economically — to adequate, safe, and nutritious food needed to meet their dietary requirements for a healthy, active, and productive life^(1,2)^. Conversely, food security is attained when every person, at all times, has reliable physical, social, and economic access to sufficient, safe, and nutritious food that satisfies their dietary needs, supporting an active and healthy lifestyle^(3,4)^.

Food security is closely linked to both nutrition security and overall health. Nutrition security is attained when a person’s body tissues receive the right amounts of nutrients and essential substances. This is achieved through a combination of household food security, access to healthcare, and meeting other basic needs such as water, hygiene, and sanitation^(5–7)^. At the household level, food insecurity occurs in two forms: chronic and transitory. Chronic food insecurity occurs when a household is unable to meet the minimum amount of food needed for a healthy life for three or more months. On the other hand, if the food insecurity is for less than 3 months, it will be transitory food insecurity^(8)^.

In 2023, an estimated 2.33 billion people, or nearly 30% of the global population, experienced moderate or severe food insecurity^(9)^. Of this number, 281.6 million individuals faced high levels of acute food insecurity^(10)^.

Globally, 31.9% of rural populations experienced moderate or severe food insecurity, compared to 29.9% in peri-urban areas and 25.5% in urban areas. In Africa, food insecurity remained largely unchanged between 2022 and 2023. However, Africa had the highest proportion of its population facing food insecurity compared to other regions, with 58.0% of the population experiencing moderate or severe food insecurity — about twice the global average — and 21.6% facing severe food insecurity. In Eastern Africa, 64.5% (313 million people) of the population faced moderate or severe food insecurity in 2023, with 24.2% severely affected^(9)^.

Ethiopia is facing severe food crises caused by ongoing droughts, macroeconomic difficulties, and internal conflicts, with extreme weather leading to livestock deaths and negatively impacting household food and nutrition security, especially in pastoral areas of Southern Ethiopia. From 2021 to 2022, the situation worsened, with acute food insecurity affecting around 19.7 million people in 2023^(10)^. In developing countries like Ethiopia, rural communities face numerous challenges in attaining food security, including fluctuations in rainfall and temperature. In 2015/16, many Ethiopians were vulnerable to drought due to food insecurity, and this issue continues to persist, particularly in pastoralist areas^(11,12)^.

The 2023 Global Hunger Index (GHI) reveals that little progress has been made in reducing hunger since 2015. The global GHI score for 2023 stands at 18.3, which is categorised as moderate^(13)^. The world remains far from achieving Sustainable Development Goals (SDGs) two, Zero Hunger. After a sharp increase in global hunger from 2019 to 2021, hunger levels have remained nearly unchanged for the past 3 years, affecting 9.1% of the population (735 million people) in 2023, up from 7.5% in 2019. Africa continues to have the highest proportion of people facing hunger, with around 300 million people (20.4%) affected in 2023, and the number continues to grow. By 2030, Africa is expected to account for 53% of the global population suffering from hunger. Eastern Africa, in particular, is home to more than half (138.5 million) of Africa’s hungry population in 2023^(9)^. According to the 2023 GHI, Ethiopia faces a serious hunger situation, with a GHI score of 26.2^(13)^.

The consequence of food insecurity is that approximately 9 million people globally die each year due to hunger and hunger-related illnesses, surpassing the combined death toll from acquired immunodeficiency syndrome (AIDS), malaria, and tuberculosis^(14)^. While hunger impacts individuals of all ages and genders, children in Africa are particularly vulnerable. Malnutrition significantly contributes to high infant mortality rates across the continent and is a leading cause of physical and cognitive developmental issues in sub-Saharan Africa (SSA)^(15)^. Beyond its direct impact on health, food insecurity also leads to disordered eating habits, fluctuating blood cholesterol levels, reduced serum albumin, lower haemoglobin, vitamin A deficiencies, and deteriorating physical and mental health^(16–18)^. Though hidden hunger predominantly affects pregnant women, children, and adolescents, it has repercussions on people’s health throughout their lives^(19)^. Given this, researchers and policymakers must prioritise addressing food insecurity and its effects on health. The SDGs, established in 2015, aim to eliminate hunger by 2030. However, the number of people experiencing hunger and food insecurity continues to rise, leading to millions of deaths. In Ethiopia, nearly one-third of the population lives below the poverty line. Consequently, chronic and acute food insecurities are widespread, particularly among rural communities and smallholder farmers. During periods of frequent drought, the proportion of chronically food-insecure individuals, which is typically 10%, can increase to over 15%^(20)^.

Achieving food security requires households to have unrestricted access to a balanced and nourishing diet. Access to such a diet primarily depends on the availability of sufficient economic resources to procure food within the country, region, district, or community where the households reside. The reasons behind food insecurity are numerous and interconnected and differ across different locations. Major contributors to food insecurity within a country encompass various factors such as poverty, the ill health of household members, suboptimal livelihoods, household management practices, natural disasters (such as climate change, droughts, and rainfall patterns), population growth, land fragmentation, land degradation, insecure land tenure, insufficient infrastructure, unemployment, low-income levels, fluctuations in agricultural commodity prices, limited access to healthcare, conflicts, and warfare. These factors can directly or indirectly impact food security within a particular area^(21,22)^.

Despite different strategies and interventions applied to tackle, food insecurity continues to be a major public health concern in developing countries like Ethiopia, particularly in rural parts of the countries like pastoralist areas. Ethiopia has struggled to make progress towards achieving SDG Target 2.1 or Target 2.2, which aims to eliminate hunger, food insecurity, and all forms of malnutrition by 2030. Exploring food insecurity from different area-specific, cultural, and socioeconomic contexts is highly demanded for evidence-based interventions and attaining SDGs. Moreover, the majority of previous studies have concentrated on agrarian communities, with limited research exploring the causes of food insecurity and the coping strategies in pastoralist areas such as the South Omo Zone. Hence, this study aimed to explore the causes of food insecurity and the coping strategies utilised by the pastoralist and agrarian communities in South Omo Zone, Ethiopia.

## Methods

### Study area

The research took place in the South Omo zone of Southern Ethiopia, situated approximately 500 kilometres (km) from Hawassa, the capital city of the Southern Region, and 750 km from Addis Ababa, the capital city of Ethiopia^(23)^. The zone is composed of 10 districts and one city administration, which is Jinka town. Out of these, six districts are pastoralists — Maale, Hammer, Salamago, Dasenech, Benatsemay, and Gnangatom — while the remaining four districts — South Ari, Woba Ari, North Ari, and Bakadawula Ari — are agrarians.

According to a report from the South Omo Zone health department, the zone contains 268 kebeles (the smallest administrative unit), of which 246 are rural and 22 are urban kebeles^(24)^. The zone exhibits a diverse range of agro-ecologies, spanning from hot arid to tropical humid climates. Its landscape comprises a minimal portion of highland (Dega) at 0.5%, midland (Woynadega) at 5.1%, semi-arid (semi-kola) at 60%, and lowland at 34.4%. The zone’s southernmost point, near Lake Rudolf, sits at the lowest altitude of about 376 m above sea level, while its highest peak, the Shengamaa Mountain in South Ari woreda, reaches approximately 3418 m above sea level in the northeastern part of the zone. Rainfall distribution varies across the zone, with lower precipitation observed towards the northeast. The average annual rainfall in the zone ranges from 400 mm to 1600 mm^(24)^.

Agriculture serves as the primary economic activity in the zone and is the predominant source of livelihood, primarily focusing on subsistence farming for personal consumption. The main crops cultivated in the area include maize, sorghum, teff, coffee, vegetables, root crops, pulses, and oilseeds. The communities in the zone are predominantly agro-pastoralists, and their livestock include cattle, goats, sheep, horses, and mules^(24)^. The study was conducted in two purposefully selected districts of the South Omo Zone: one from the agrarian communities of the zone (South Ari woreda) and one from pastoralist communities of the zone (Benatsemay woreda).

### Population

In this study, health extension workers, health centre directors, woreda nutrition programme experts (health and agriculture), and district health managers were included in the detailed interviews. Additionally, pregnant women staying in maternity waiting homes at health facilities participated in the focus group discussions.

### Sample size determination

Generally, there is no set of rules or exact criteria provided to determine the sample size in qualitative research. In this study, the researcher intentionally chose participants until reaching the stage of idea saturation, guaranteeing thorough gathering of information. Attaining data saturation is widely recognised as a methodological principle in qualitative research, indicating that once enough data have been gathered, further collection or analysis is unnecessary^(25,26)^.

Data saturation depends on the depth of information provided by participants regarding the research problem. A total of 17 participants underwent in-depth interviews, while two focus group discussions involved 27 pregnant women staying at maternity waiting homes at health facilities, with a total of 12 and 15 participants per group, respectively. Through these interviews and discussions, we gathered a range of perspectives and suggestions on household food security.

### Sampling method and sampling procedure

The concept of sampling method in qualitative research encompasses a range of approaches tailored for analysing data presented in natural language, including verbal expressions and the portrayal of experiences encountered in social interactions and artistic forms^(27,28)^. For this study, a purposive sampling method was utilised to choose participants, focusing specifically on health extension workers, health centre directors, woreda programme experts (health and agriculture office), district health managers, and pregnant women staying in maternity waiting homes.

The selection process involved deliberately choosing one health facility from each district, specifically targeting those with the highest number of pregnant women staying in maternity waiting homes. Therefore, Kako Health Center in Benatsemay district, which accommodates 20–30 pregnant women on average monthly, was intentionally selected. Likewise, Gazer Primary Hospital, which accommodates 25–35 pregnant women per month in maternity waiting homes, was deliberately selected for focus group discussions (FGD). The researcher intentionally chose participants until reaching the stage of idea saturation.

### Data collection tool, personnel, and procedure

In this study, two distinct qualitative research approaches, namely in-depth interviews and focus group discussions (FGD), were utilised for gathering the data for the study. A discussion guide for the FGD, comprising open-ended probing inquiries, was utilised as the instrument for data collection. Conversely, the key informant interview guide, which also included open-ended probing questions, was used to gather information.

The focus group discussions were conducted in the Benegna language at Kako Health Center, while at Gazer Primary Hospital, they were conducted in Amharic, the official language used in both the South Omo Zone and Ethiopia. In-depth interviews with health extension workers, health center directors, woreda program experts (health and agriculture office), were also conducted by the researcher in Amharic.

During the data collection, focus group discussions and in-depth interviews were recorded, and field notes were taken on the discussions. Consequently, the recorded interview data were transcribed at the end of each day. In the current research, special precaution and practice measures were implemented to control the spread of COVID-19. The assurance of data quality was achieved by taking into account criteria for trustworthiness such as confirmability, transferability, dependability, and credibility^(29–32)^.

### Data management and analysis

According to Creswell and Creswell, the data analysis outlines the analytic approach employed in the study^(26)^.

In this study, the researcher adopted an interactive and iterative analytic approach with multiple stages, organised in a logical order. The collected data, including field notes, audio records, and interview audio, were prepared for analysis. This involved transcribing the data and gaining an overall understanding of the content through an initial analysis. General ideas were documented in notes, forming the basis for further thematic analysis.

The thematic analysis involves several steps, as articulated by the eight coding process steps: reading through the data carefully, selecting a standout document, listing all themes, grouping related themes in a table, coding next to the topics, describing and reducing themes, making final decisions on abbreviations, arranging subthemes in alphabetical order, grouping data within each category, and conducting preliminary analysis.

## Results

### Biographical profile of key informants

A total of 17 participants which included health extension workers, district health managers, health centre/hospital directors, and woreda programme experts (health and agriculture) participated in the interview. Most of the study participants, 82.4% (*n*=14) were females and 17.6% (*n*=3) were males. More than two-thirds of the participants, 70.6% (*n*=12), were in the age range of 31–40 years. The median age of the participants was 37 years with a range of 29 to 41 years.

Most of the participants, 70.6% (*n*=12), were qualified for a diploma, and 29.4% (*n*=5) participants had a degree qualification. More than half of the participants, 52.8% (*n*=9), were health extension workers, 11.8% (*n*=2) of participants were district agriculture experts, 11.8% (*n*=2) of participants were woreda health experts, 11.8% (*n*=2) participants were district health heads, and the rest 11.8% (*n*=2) of the participants were health centre and hospital directors (Table 1).

**Table 1.** Characteristics of in-depth interview participants in South Omo Zone, Southern Ethiopia, 2023 (*n*=17)

Variable	Frequency	Percent
Sex		
Male	3	17.6
Female	14	82.4
Age		
20–30	3	17.6
31–40	12	70.6
≥ 41	2	11.8
Qualification		
Diploma	12	70.6
Degree	5	29.4
Responsibility		
Health extension workers	9	52.8
Health centre/hospital directors	2	11.8
Woreda agriculture experts	2	11.8
Woreda health experts	2	11.8
District health managers	2	11.8

The researcher identified three main themes and their corresponding subthemes based on the results, which include the burden of food insecurity, causes of food insecurity, and coping strategies for managing food insecurity at household levels.

### The burden of food insecurity

Despite government efforts to address food insecurity, it remains a significant global concern, especially in developing nations like Ethiopia. The participants unanimously confirmed the presence of food insecurity in the study area.

A 38-year-old district manager and key informant stated that: “…. the economic burden on the pastoralist community has been exacerbated by food insecurity”.

A 36-year-old nutrition focal person from a pastoralist woreda reported that: “…43,354 people in two clusters of the woreda are receiving food aid from the government this year”. Additionally, a 39-year-old female participant from another semi-pastoralist community explained that: “…. drought has caused significant livestock loss in their area, worsening food insecurity”.

### Causes of food insecurity

In the study, participants in both the in-depth interviews and focus group discussions frequently identified several causes of food insecurity in the community: food shortages, climate change, rising agricultural product prices, and inadequate agricultural technology.

#### Shortage of food

The respondents in the interviews and focus group discussions were specifically asked about the factors leading to food insecurity in the study area. A common consensus among the participants was that food shortages were a major issue. They pinpointed the lack of food availability and access as the main reasons for food insecurity in the area. This indicates that the participants see food insecurity as stemming from both an inadequate supply of food and difficulties in accessing food.

A 38-year-old man, key informant, said:

The main factor contributing to food insecurity in this area is the insufficient availability of food resulting from the prolonged dry season.

#### Climate change

Participants highlighted climate change as a significant factor contributing to food insecurity, specifically emphasising on shortage of rain and the occurrence of drought in the study areas.

A 30-year-old woman, a key informant said that:

…I recall a significant loss of livestock in our area during the last drought.

This shows the impact of climate-induced events, such as drought, on the agricultural and livestock sectors, thereby exacerbating food insecurity in the community.

#### Inflation of price of agricultural products

The participants emphasised the presence of inflation in food products within the area. Through focus group discussions and interviews, it was discovered that the inflation of agricultural products, particularly food items, is linked to the community’s pastoralist lifestyle. The community often sells food products before the harvest season, resulting in ongoing food insecurity. This habit of selling agricultural goods prematurely contributes to rising food prices, worsening the community’s difficulties in achieving food security.

A 29-year-old woman, a key informant, explained that:

…Pastoralists face a vulnerability as they sell their limited agricultural products on the farmland before the harvest, representing a notable weakness in their economic approach. Subsequently, they are compelled to sell animal products to procure essential food items for their families, such as seeds, vegetables, and fruits. However, this transaction occurs amid price inflation, placing an additional economic burden on the pastoralist community.

#### Lack of agricultural technology

Participants emphasised that the utilisation of traditional farming methods is a significant factor contributing to low agricultural production, ultimately resulting in food shortages.

A 41-year-old woman, a key informant, explained that:

…I think that the government’s backing of the settlement project for pastoralist communities is beneficial in terms of supporting irrigation and the transfer of agricultural technology.

A 34-year-old man, key informant, said that:

…Farmers are using a traditional cultivation system, which is rain-based. It is not modernized yet. Food insecurity happens also due to the rain-fed agriculture system. Hence, they have nothing to save for the future. Some of the farmers finish the products before harvest.

### Coping strategies for managing food insecurity

During in-depth interviews, many participants discussed how the community manages their food insecurity issues. These included selling livestock such as cows, cattle, or goats, minimising the frequency of meals, limiting the diversity of food options, relying on food assistance, forgoing personal meals to prioritise children’s nutrition, and relocating to other areas in search of sustenance.

In this study, minimising the frequency of meals was consistently identified as one of the coping strategies for managing food insecurity.

A 41-year-old man, key informant, explained that:

For individuals with stable employment, such as myself, receiving a consistent salary allows the possibility of streamlining daily meals from four (breakfast, lunch, dessert, and supper) to three (breakfast, lunch, and supper). However, the situation is more challenging for rural communities, as their lack of regular income makes it difficult to navigate food shortages. These individuals encounter difficulties in setting aside savings for the future because they lack a stable source of income.

This finding highlights the impact of employment on the food security of households is noteworthy for both vulnerable and secure households. Moreover, its influence becomes more pronounced as the quantiles of food security rise.

Selling livestock such as cows, cattle, or goats is found as one of the coping strategies.

A 39-year-old woman, an FGD participant, explained that,

When there is a food shortage, families sell their livestock to buy food supplies.

A 29-year-old woman, a key informant, explained that:

…Individuals with financial resources can make purchases from the marketplace, while others have the option to move to other places in search of food. Those possessing assets at home also have the opportunity to sell them and acquire food.

This finding indicates that the sale of livestock, a commonly owned asset among both pastoralist and non-pastoralist households, is one of the predominant strategies used. Engaging in such urgent sales diminishes the asset reserves of households, thereby compromising their prospective capacity to rebound from unforeseen challenges.

Another commonly adopted tactic was the widespread reliance on support from friends and family, which involved receiving financial assistance for purchasing food and borrowing money. Sending livestock in search of pasture, engaging in new wage labour, migrating certain family members, and seeking loans from savings or microfinance institutions are also commonly used strategies to cope with food insecurity.

A 34-year-old man, key informant, explained that:

The issue of household-level food insecurity in pastoralist areas is highly critical, with the entire community depending on assistance provided by the Agriculture, Food Security and Preparedness Office for survival.

This finding found that being a beneficiary of a productive safety net programme is one of the means to cope with the effects of food insecurity.

## Discussion

Despite different efforts to address food insecurity, it remains a significant global concern, especially in developing nations like Ethiopia. The number of people affected by food insecurity increased to 815 million in 2016, up from 777 million in 2015 and 775 million in 2014^(33,34)^. The participants unanimously confirmed the presence of food insecurity in the study area.

A 38-year-old district manager and key informant stated that: “…. the economic burden on the pastoralist community has been exacerbated by food insecurity.” This finding implies that the economic burdens faced by pastoralists are closely linked to food insecurity. When pastoralists cannot afford to maintain their herds due to economic pressures, their ability to produce food diminishes. This creates a vicious cycle where economic hardship leads to food scarcity, which in turn exacerbates poverty and vulnerability^(35)^.

A 36-year-old nutrition focal person from a pastoralist woreda reported that: “…43,354 people in two clusters of the woreda are receiving food aid from the government this year”. Additionally, a 39-year-old female participant from another semi-pastoralist community explained that: “…. drought has caused significant livestock loss in their area, worsening food insecurity”.

Pastoralist communities often face challenges such as drought, fluctuating market prices, and limited access to resources, which can lead to inadequate food availability^(36)^. Pastoralists typically rely on livestock for their livelihoods. However, economic pressures — such as loss of livestock due to disease or climate change — can significantly impact their ability to secure food. Moreover, climate change exacerbates the challenges faced by pastoralists, as erratic weather patterns can lead to diminished grazing land and water sources. This results in reduced livestock productivity and increased reliance on external food aid^(37,38)^. The FAO emphasises that climate-related shocks disproportionately affect vulnerable populations, including pastoralists^(35)^. The reliance on food aid indicates a need for targeted interventions that address both immediate food needs and long-term resilience strategies. Programmes aimed at improving livestock health, diversifying income sources, and enhancing access to markets can help reduce dependency on aid.

The current study attested that food shortages, climate change, unmet family planning needs, rising agricultural product prices, and inadequate agricultural technology studies are causes of food insecurity in the community.

Participants in the interviews and focus group discussions were specifically asked about various factors that contribute to food insecurity in the study areas. The participants agreed that food shortages were a major concern. They identified a lack of food availability and access as the main driver of food insecurity in the area. This suggests that the participants believe food insecurity is caused by both a scarcity of food and challenges with accessing it.

A 38-year-old man, key informant, said:

The main factor contributing to food insecurity in this area is the insufficient availability of food resulting from the prolonged dry season.

This finding is substantiated by the Food and Agriculture Organization’s (FAO) research, which indicates that climate change negatively influences food availability by significantly impacting crop yields, fish population, and the health and productivity of animals. This impact is particularly pronounced in regions such as sub-Saharan Africa, where a substantial proportion of the current population experiencing food insecurity resides^(21)^.

In this study, participants highlighted climate change as a significant factor contributing to food insecurity, specifically emphasising on shortage of rain and the occurrence of drought in the study areas.

A 30-year-old woman, a key informant said that:

…I recall a significant loss of livestock in our area during the last drought.

This shows the impact of climate-induced events, such as drought, on the agricultural and livestock sectors, thereby exacerbating food insecurity in the community.

This finding was supported by findings of the world’s FAO that Climate change presents a significant and escalating challenge to food security. Anticipated consequences of climate change, such as elevated temperatures, increased occurrence of extreme weather events, water scarcity, rising sea levels, ocean acidification, land degradation, disruption of ecosystems, and decline in biodiversity, hinder efforts to eliminate hunger, malnutrition, and poverty^(21)^.

The participants emphasised the presence of inflation in food products within the area. Through focus group discussions and interviews, it was discovered that the inflation of agricultural products, particularly food items, is linked to the community’s pastoralist lifestyle. The community often sells food products before the harvest season, resulting in ongoing food insecurity. This habit of selling agricultural goods prematurely contributes to rising food prices, worsening the community’s difficulties in achieving food security.

A 29-year-old woman, a key informant, explained that:

…Pastoralists face a vulnerability as they sell their limited agricultural products on the farmland before the harvest, representing a notable weakness in their economic approach. Subsequently, they are compelled to sell animal products to procure essential food items for their families, such as seeds, vegetables, and fruits. However, this transaction occurs amid price inflation, placing an additional economic burden on the pastoralist community.

This result was supported by various studies^(39,40)^ indicating that, in periods of elevated commodity prices, individuals with higher wealth can improve their social standing. Conversely, rural smallholder farmers do not experience comparable advantages owing to their production of smaller quantities. This illustrates the impact of price shocks, such as the inflation of agricultural product prices, on food security, highlighting the connection to individuals’ economic status^(41)^. The relationship between price inflation and food insecurity is complex and multifaceted. Understanding these dynamics is crucial for policymakers aiming to mitigate the impacts of inflation on vulnerable populations and ensure food security. This underscores the need for policies that address both food affordability and economic stability.

Participants emphasised that the utilisation of traditional farming methods is a significant factor contributing to low agricultural production, ultimately resulting in food shortages.

A 41-year-old woman, a key informant, explained that:

…I think that the government’s backing of the settlement project for pastoralist communities is beneficial in terms of supporting irrigation and the transfer of agricultural technology.

This finding was supported by research findings that emphasised the positive impact of irrigation, whether it is complete or supplementary. Irrigation was found to decrease dependence on unpredictable rainfall and minimise the impacts of droughts, resulting in increased agricultural yields. Additionally, irrigation extends cropping time and cycles, enables the cultivation of a broader range of crops, and provides stable conditions for the application of fertilizers as an additional method to enhance overall yield^(42)^.

A 34-year-old man, key informant, said that:

…Farmers are using a traditional cultivation system, which is rain-based. It is not modernized yet. Food insecurity happens also due to the rain-fed agriculture system. Hence, they have nothing to save for the future. Some of the farmers finish the products before harvest.

This finding aligns with Nawaiseh’s research, which advocates for the broader incorporation of cutting-edge technology in agriculture and advanced communication methods. This approach aims to disseminate knowledge and enhance awareness among farmers, ultimately leading to heightened production, enhanced quality, and more efficient water utilisation^(43)^.

### Practical implications

This study has significant implications for programmes and policies aimed at improving food security. Addressing food insecurity in agrarian and pastoralist communities requires a multifaceted approach that takes into account the unique socio-economic and environmental contexts of these populations. This finding highlights the need to implement targeted interventions, enhance resource management, promote education, improve market access, establish social safety nets, and develop integrated policy frameworks to make significant strides toward alleviating food insecurity in the South Omo Zone and similar regions.

### Limitations of the study

This study has some limitations. The study was conducted in two districts of the South Omo Zone and may not be easily generalisable to other pastoralist and agrarian areas or populations due to the specific context of the South Omo Zone. Moreover, participants may provide socially desirable responses rather than sharing their true experiences, especially regarding sensitive topics like food insecurity.

### Conclusion

This study revealed that the causes of food insecurity include food shortages, climate change, lack of awareness, scarcity of farmland, inadequate farming technology, and income constraints. Hence, tailored strategies to implement policies for stabilising food supply chains and mitigating food shortages in the agrarian and pastoralist areas are highly demanding. Investing in modern agricultural technologies to boost productivity, encourage the adoption of climate-smart agricultural practices to help farmers adapt to changing weather patterns, and optimise the productive use of available farmland are also highly needed. Moreover, a productive safety net programme is required to improve household income.
